# Fertility Complications After Uterine Artery Embolization for Postpartum Hemorrhage: A Case of Infertility and Miscarriage Following Stillbirth

**DOI:** 10.7759/cureus.82609

**Published:** 2025-04-19

**Authors:** Hiroaki Hiraga, Kyohei Hoshino, Emi Yokoyama, Yasuno Takahashi, Takeki Sato, Yuri Takahashi, Jumpei Toratani, Akira Kuga, Aiko Takahashi, Zen Watanabe, Masahito Tachibana, Masatoshi Saito

**Affiliations:** 1 Department of Obstetrics and Gynecology, Tohoku University Hospital, Sendai, JPN

**Keywords:** endometrial thinning, fertility preservation, infertility, miscarriage, postpartum hemorrhage, uterine artery embolization

## Abstract

Uterine artery embolization (UAE) is widely used as a minimally invasive treatment for postpartum hemorrhage (PPH), with the expectation of preserving fertility. However, its effects on fertility remain unclear. We present the case of a 32-year-old woman who developed secondary infertility after undergoing UAE for severe PPH following a stillbirth at 37 weeks of gestation. Despite the resumption of regular menstruation, the endometrium remained persistently thin, and hysterosalpingography revealed narrowing of the uterine cavity. She underwent assisted reproductive technology (ART), but repeated frozen-thawed embryo transfer (FET) cycles resulted in implantation failure. Although she eventually conceived, she experienced a missed abortion at seven weeks. No specific causes for the miscarriage were identified. This case highlights that even in the absence of intrauterine adhesions or amenorrhea, endometrial thinning and uterine cavity narrowing after UAE can contribute to infertility and miscarriage. UAE should be carefully considered in women desiring future pregnancies, and alternative hemostatic measures should be prioritized when possible. Further research is required to improve UAE techniques and fertility preservation.

## Introduction

Postpartum hemorrhage (PPH) is the leading cause of maternal mortality [1]. The causes of PPH are often categorized using “four Ts”: uterine atony (tone), birth canal laceration (trauma), retained placenta and placental abnormalities such as placenta accreta spectrum (tissue), and coagulation disorders (thrombin) [1]. Initial management involves assessing blood loss, stabilizing vital signs through fluid resuscitation and transfusion, administering uterotonic agents, and performing uterine fundal massage. Subsequent interventions, such as balloon tamponade, uterine compression sutures, manual removal of the placenta, laceration repair, and treatment for disseminated intravascular coagulation, are performed based on the underlying cause.

Hysterectomy has traditionally been the last resort for uncontrolled PPH. However, in recent years, uterine artery embolization (UAE) has become an increasingly popular minimally invasive treatment that does not require laparotomy or general anesthesia. In Japan, more than 100 medical institutions offer UAE for PPH [2]. UAE has several advantages over hysterectomy, including reduced blood loss, shorter procedure time, and a shorter hospital stay [3]. It also preserves the uterus, making fertility preservation possible. There have been many reports of successful deliveries following UAE [4,5].

In contrast, UAE for PPH is associated with an increased risk of placenta accreta and recurrent PPH in subsequent pregnancies [6]. Additionally, complications such as intrauterine adhesions and premature ovarian insufficiency can lead to amenorrhea and infertility [7-9]. However, the potential contribution of uterine ischemia-induced endometrial thinning to infertility and miscarriage remains unclear.

This report presents a patient who underwent UAE for PPH following stillbirth. Despite the resumption of menstruation, she developed endometrial thinning, which was strongly suspected to have contributed to infertility and miscarriage.

## Case presentation

A 32-year-old woman was referred to Tohoku University Hospital, Japan, with suspected endometrial thinning after 10 months of attempting conception. She was gravida 1, para 1 (G1P1) and had conceived spontaneously at the age of 29. The pregnancy was managed at a local clinic and progressed without complications; however, she experienced a stillbirth at 37 weeks of gestation (birth weight: 2812 g) at the age of 30. The cause was presumed to be excessive umbilical cord twisting.

**Figure 1:**
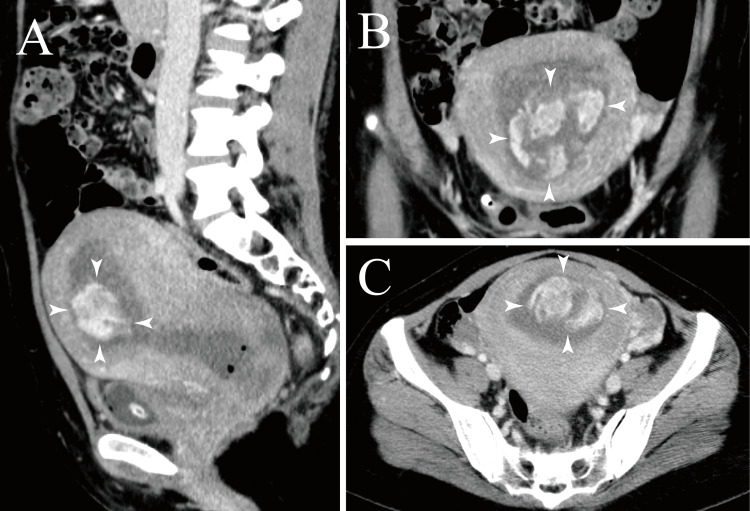
Contrast-enhanced computed tomography image before uterine artery embolization at a regional hospital Computed tomography shows a retained placenta and extravasation (arrowheads) into the uterine cavity. A) Sagittal view; B) Coronal view; C) Axial view

Thirteen hours postpartum, she experienced significant hemorrhage, losing 1270 mL of blood, and the bleeding persisted. On postpartum day 3, ultrasonography revealed a retained placenta, prompting a transfer to a regional hospital. The patient had no history of uterine fibroids or coagulation disorders. She developed anemia with a hemoglobin level of 4.7 g/dL, necessitating the transfusion of 8 units of red blood cells and 2 units of fresh frozen plasma. Computed tomography revealed extravasation in the uterine cavity (Figure 1). Consequently, bilateral UAE was performed using gelatin sponge fragments prepared by pumping. On postpartum day 5, after confirming by ultrasonography that there was no residual blood flow in the placenta and no findings suggestive of placenta accreta, 130 g of the retained placenta was removed using placental forceps. Her white blood cell count rose to 16.36 × 10⁹/L, and C-reactive protein levels peaked at 244.6 mg/L, suggesting an intrauterine infection. The patient was treated with intravenous cefmetazole, followed by oral amoxicillin/clavulanic acid, and was discharged on postpartum day 8.

Her menstruation resumed regularly in a 29-day cycle, with flow subjectively reduced from pre-pregnancy levels. Following her physician’s advice, she avoided pregnancy for one year postpartum. She did not use oral contraceptives, a contraceptive implant, or an intrauterine device during this period. At the age of 31 years, she began trying to conceive but was unable to after eight months. She visited a local gynecological clinic at the age of 32 years, where evaluation revealed a small uterus and a thin endometrium measuring only 5 mm in the secretory phase. The physician determined that specialized reproductive treatment was necessary and referred the patient to our hospital’s reproductive medicine clinic.

**Figure 2:**
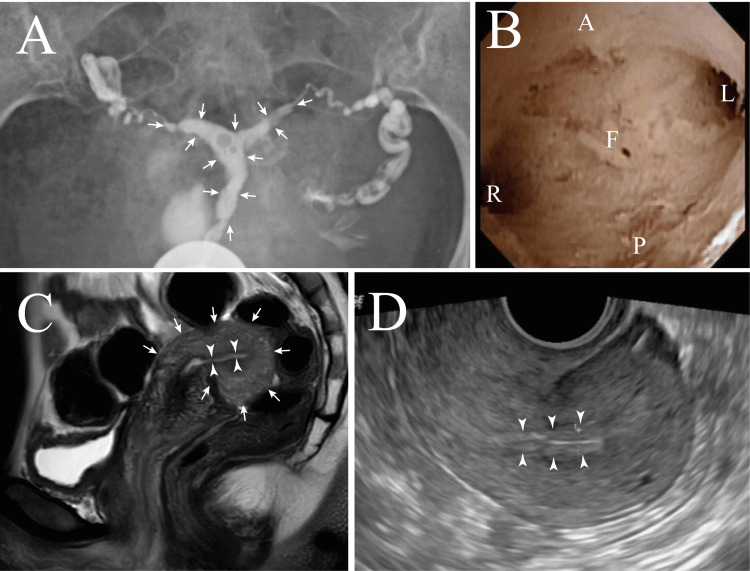
Imaging findings at the initial treatment stage at our hospital A) Hysterosalpingography showing poor expansion of the uterine cavity (arrows) despite contrast injection, suggesting decreased myometrial compliance. B) Hysteroscopy showing no intrauterine adhesions. A: anterior wall; P: posterior wall; R: right tubal ostium; L: left tubal ostium; F: fundus C) MRI showing uterine atrophy (arrows) and endometrial thinning (arrowhead). D) Transvaginal ultrasonography on day 12 of the first cycle with letrozole; follicles of 13 mm (right ovary), 21 mm, and 15 mm (left ovary) were observed. Despite adequate estradiol production, the endometrium remained thin at 4.0 mm (arrowheads).

Infertility screening was performed, and laboratory tests revealed no abnormalities in basal hormone levels, luteal phase progesterone, anti-Müllerian hormone, thyroid function, or Chlamydia trachomatis antibody levels (Table 1). The results of her husband's semen analysis were normal (Table 2). Ultrasonography, hysterosalpingography, and magnetic resonance imaging (MRI) revealed a small uterine body, thin endometrium, and narrowed uterine cavity (Figure 2). The fallopian tubes were normal, and hysteroscopic examination revealed no intrauterine adhesions. Endometrial thinning was suspected to be the cause of secondary infertility.

**Table 1:** Laboratory findings from infertility screening LH: luteinizing hormone; FSH: follicle-stimulating hormone; TSH: thyroid-stimulating hormone; fT4: free thyroxine; fT3: free triiodothyronine; IgA: immunoglobulin A; IgG: immunoglobulin G

Laboratory parameters		Patient values	Reference range
Basal hormone levels (early follicular phase, days 1-5)	FSH (mIU/mL)	5.59	3.5-12.5
	LH (mIU/mL)	5.91	<10.0
	Estradiol (pg/mL)	63.5	11-82
	Prolactin (ng/mL)	18.1	5-30
	Testosterone (ng/dL)	7	11-47
Luteal phase progesterone (ng/mL)		32.2	>10
Anti-Müllerian hormone (ng/mL)		2.41	0.62-13.87 (for age 32)
Thyroid function	TSH (mIU/L)	1.760	0.50-5.00
	fT4 (ng/dL)	1.39	0.90-1.70
	fT3 (pg/mL)	2.58	2.30-4.00
Chlamydia trachomatis antibody	IgA (index)	0.38	<0.90
	IgG (index)	0.79	<0.90

**Table 2:** Semen analysis

Laboratory parameters	First test	Second test	Reference range
Semen volume (mL)	5.5	5.0	>1.4
Sperm concentration (million/mL)	93.3	50.8	>16
Total sperm count (million)	513	254	>39
Total motility (%)	71	81	>42

Ovarian stimulation was initiated using an aromatase inhibitor, and uterine blood flow was improved with tocopherol acetate, unkei-to (a Kampo medicine used to improve blood circulation and warm the uterus), and low-dose aspirin. Two cycles of timed intercourse and one cycle of intrauterine insemination were attempted, but none resulted in pregnancy. The patient opted for assisted reproductive technology (ART).

At the age of 33, she underwent her first oocyte retrieval, which yielded 10 oocytes. However, only one was fertilized normally using conventional in vitro fertilization, and no blastocysts were obtained. During the second retrieval, 11 oocytes were obtained, and intracytoplasmic sperm injection (ICSI) resulted in seven fertilized embryos. One blastocyst classified as Gardner grade 4AA was cryopreserved. A frozen-thawed embryo transfer (FET) in a hormone replacement cycle (HR-FET) was performed (Table 3). However, her endometrium remained thin, and the serum human chorionic gonadotropin (hCG) test on post-embryo transfer day 8 was negative.

**Table 3:** Details of embryo transfer Low-dose aspirin, unkei-to, and tocopherol acetate were used for all embryo transfers. hCG: human chorionic gonadotropin

Embryo transfer	Estradiol administration	Endometrial thickness		Embryo	Hyaluronic acid-enriched transfer medium	Outcome
		before progesterone administration	on transfer day			
1st	Transdermal patch 2.88 mg/2 day	4.3 mm	4.4 mm	4AA	+	Serum hCG negative
2nd	Oral tablet 6 mg/day	4.7 mm	3.6 mm	4BA	+	Serum hCG negative
3rd	Transdermal patch 2.88 mg/2 day	4.7 mm	4.2 mm	5BB	+	Missed abortion at 7 weeks/normal chorionic karyotype

During the third oocyte retrieval, 18 oocytes were obtained, and 12 embryos were fertilized via ICSI. Two blastocysts classified as Gardner grade 4BB or 4BA were cryopreserved. At the age of 34, she underwent a second HR-FET. Her endometrium remained thin, and pregnancy was not achieved (Table 3). Endometrial receptivity analysis, endometrial microbiome testing, screening for chronic endometritis, and referral to a facility offering platelet-rich plasma (PRP) therapy or granulocyte colony-stimulating factor (G-CSF) therapy were suggested. However, because these treatments were not covered by Japan’s public health insurance, the patient declined them.

Her endometrium remained thin at the third HR-FET, but she conceived successfully. Nine days after embryo transfer (gestational week 4+1), her serum hCG level was 338 mIU/mL. A gestational sac was confirmed at five weeks of gestation, and fetal heart activity was detected at six weeks. However, at seven weeks of gestation, she was diagnosed with a missed abortion. Chromosomal analysis of chorionic villi revealed a normal karyotype (Table 3).

Three months after the miscarriage, blood tests were conducted to measure antinuclear antibodies, lupus anticoagulant, factor XII activity, protein C activity, protein S activity, anti-cardiolipin antibodies, anti-beta-2 glycoprotein I antibodies, and activated partial thromboplastin time (Table 4). All results were within the normal range and no findings indicating the cause of miscarriage were identified. Ultimately, she decided to discontinue infertility treatment.

**Table 4:** Laboratory findings from miscarriage screening NR: normalized ratio; IgG: immunoglobulin G; IgM: immunoglobulin M; β2: beta-2

Laboratory parameters		Patient values	Reference range
Antinuclear antibodies titer		<1:40	<1:80
Lupus anticoagulant (NR)		0.98	<1.2
Factor XII activity (%)		90	46-156
Protein C activity (%)		106	64-135
Protein S activity (%)		78	64-149
Anti-cardiolipin antibodies	IgG (U/mL)	5.7	<20
	IgM (U/mL)	5.8	<20
Anti-β2 glycoprotein I antibodies	IgG (U/mL)	9.7	<20
	IgM (U/mL)	3.4	<20
Activated partial thromboplastin time (second)		25.6	24.3-34.6

## Discussion

UAE for PPH is widely used due to its advantages over hysterectomy, including reduced blood loss, shorter operative time, and shorter hospital stays [3]. Given its minimally invasive nature and potential for fertility preservation, UAE has become a particularly prevalent alternative to hysterectomy in Japan. However, a known complication is an increased risk of placenta accreta and recurrent PPH in subsequent pregnancies [6]. Despite its widespread use, the effect of UAE on fertility remains insufficiently studied [5,8-10]. This case suggests that endometrial thinning following UAE may contribute to infertility and miscarriage, warranting further investigation to clarify the relationship between UAE, endometrial thinning, and fertility outcomes.

In ART, a thin endometrium is associated with lower clinical pregnancy and live birth rates [11,12]. In this case, the significant degree of uterine atrophy indicated that the ischemia caused by UAE was severe. It has been hypothesized that uterine ischemia leads to endometrial thinning, which impairs fertility. Additionally, while a relatively high number of oocytes were retrieved in this case, the number of blastocysts obtained was low. Hypoxic conditions in vivo impair embryonic development [13], and UAE-induced ovarian hypoxia may result in oocyte damage, reducing embryonic developmental potential.

Although there are few studies on endometrial thinning caused by UAE, research on uterine necrosis - another ischemic complication - may provide valuable insights [2,14]. The use of gelatin sponge for embolization is generally considered safe [8], and fragments of gelatin sponge were used in this case. However, the use of microembolic agents such as gelatin sponge powder has been linked to an increased risk of uterine necrosis [14]; this was not the case for our patient. The method used to prepare gelatin sponge fragments can also impact embolization. The pumping method produces a higher proportion of smaller particles, while the cutting method results in more uniform particle sizes [15]. Consequently, Japanese guidelines recommend the cutting method over the pumping method [2]. Future research should carefully evaluate the type, preparation method, and quantity of embolic agents used in UAE procedures.

Treatments for endometrial thinning are actively being studied in reproductive medicine. Recent meta-analyses suggest that PRP therapy [16] and G-CSF therapy [17] may improve endometrial thickness and clinical pregnancy rates.

While recent studies have suggested that UAE offers advantages over hysterectomy in treating PPH, particularly due to its minimally invasive nature and strong hemostatic efficacy, the effect of UAE on fertility remains unclear [2-10]. Careful consideration of its indications is essential, especially for women who may desire future pregnancies. In these cases, low-invasive alternatives - such as maximizing the use of multiple uterotonic agents, performing bimanual uterine compression, using balloon tamponade, applying uterine compression sutures, and aggressively administering blood transfusions while attempting to remove retained products of conception - should be prioritized. As demonstrated in this case, some patients may not achieve live births after UAE. Therefore, UAE should be viewed as an alternative to hysterectomy rather than a definitive fertility-preserving treatment. Further research is required to refine UAE techniques to better preserve fertility potential.

## Conclusions

This case highlights that even in the absence of intrauterine adhesions or amenorrhea, endometrial thinning after UAE for PPH can lead to infertility and miscarriage. Although UAE is expected to preserve fertility, it may contribute to infertility and pregnancy loss. When managing PPH, hemostatic methods such as uterotonic agents, bimanual uterine compression, balloon tamponade, and uterine compression sutures should be prioritized. If vital signs can be stabilized with transfusion and fertility preservation is a high priority, UAE should be considered as a last resort, similar to laparotomic hysterectomy.
